# How different domains of quality of life are associated with latent dimensions of mental health measured by GHQ-12

**DOI:** 10.1186/s12955-021-01892-9

**Published:** 2021-11-14

**Authors:** Fatemeh Nouri, Awat Feizi, Hamidreza Roohafza, Masoumeh Sadeghi, Nizal Sarrafzadegan

**Affiliations:** 1 Student Research Committee, School of Health, Isfahan University of Medical Sciences, Isfahan, Iran; 2 Department of Biostatistics and Epidemiology, School of Health, Isfahan University of Medical Sciences, Hezar Jerib Street, 8174673461 Isfahan, Iran; 3 Isfahan Cardiovascular Research Center, Cardiovascular Research Institute, Isfahan University of Medical Sciences, Isfahan, Iran; 4 Cardiac Rehabilitation Research Center, Cardiovascular Research Institute, Isfahan University of Medical Sciences, Isfahan, Iran; 5 Heart Failure Research Center, Cardiovascular Research Institute, Isfahan University of Medical Sciences, Isfahan, Iran

**Keywords:** General Health Questionnaire (GHQ-12), Mental health, Item Response Theory, Quality of life (QoL)

## Abstract

**Background and objectives:**

A short form of the General Health Questionnaire (GHQ-12) is a useful screening instrument for assessing mental health. Furthermore, Quality of life (QoL) is a critical treatment outcome in many clinical and health care research settings. This study aimed to reassess the dimensionality of GHQ-12 using Multidimensional Graded Response Model (MGRM) and evaluate how its extracted dimensions are associated with the QoL's domains.

**Methods:**

Isfahan Cohort Study 2 (ICS2) is a population-based, ongoing prospective cohort study among adults aged 35 years and older who were free of cardiovascular diseases (CVDs) at the beginning of the study in 2013. A total of 1316 participants, all living in urban and rural areas of Isfahan and Najafabad, Iran was completed the GHQ-12 and WHO QoL-brief version at baseline. Five competing MGRMs with different latent structures were specified for GHQ-12. Factor scores derived from the best fitted model were used to associate with various domains of QoL. Results: The Three-Dimensional model for GHQ-12 was the best-fitted model explaining the Social Function (SF), Self Confidence (SC), and Anxiety/Depression (A/D) as three correlated yet different latent dimensions of mental health. Our findings in full adjusted multivariate regression models showed that a one-SD increase in dimensions of SC and SF was associated with a 38- to 48%-SD and 27- to 38%-SD increase in the domains scores of QoL, respectively. Moreover, for each one‐SD increase in score of A/D dimension, the domains scores of QoL decreased by 29- to 40%-SD. The highest to the lowest standardized coefficients for all latent dimensions of mental health were respectively related to the psychological, physical health, social relationships, and environmental condition domains of QoL. Furthermore, SC, A/D, and SF dimensions of GHQ-12 showed the highest to the lowest degree of association with all domains of QoL.

**Conclusions:**

Our findings confirm that the GHQ-12 as a multidimensional rather than unitary instrument measures distinct dimensions of mental health. Furthermore, all aspects of QoL changed when the intensity of latent dimensions of mental health increased. Moreover, the psychological domain of QoL is the most affected by all latent dimensions of mental health, followed by physical health, social relationships, and environmental condition domains. It seems that in an attempt to full recovery as assessed by improved QoL outcomes, treatment of clinical symptoms may not be sufficient. Identifying and differentiating the structures of mental health in each community as well as implementing intervention programs aimed at focusing on specific dimensions may help in the prevention of further deterioration of mental health and improved QoL in the community.

**Supplementary Information:**

The online version contains supplementary material available at 10.1186/s12955-021-01892-9.

## Introduction

Quality of life (QoL) as a critical treatment outcome is an essential topic in mental health care and research [1]. According to the World Health Organization (WHO), QoL is “as an individual's perception of his/her position in life in the context of the culture and value systems in which he/she lives and in relation to his/her goals, expectations, standards and concerns” [2]. QoL as a multidimensional construct of objective and subjective factors comprises the individual's physical health, social relationships, psychological status, and environmental conditions [2]. Previous studies showed that almost all of the QoL aspects are vulnerable to mental health status. Patients with mental disorders such as depression, anxiety, and distress have weak QoL [3–11].

According to the WHO, mental health as a critical component of general health is “a state of well-being in which the individual realizes his or her abilities, can cope with the normal stress of life, can work productively and fruitfully, and is able to make a contribution to his or her community” [12]. The General Health Questionnaire (GHQ) is a member of the family of instruments for assessing the mental aspect of general health. It evaluates symptoms of anxiety, social dysfunction, loss of confidence, and depression [13]. Of the various versions of GHQ, e.g., the GHQ-60, GHQ-30, GHQ-28, and GHQ-12, the twelve-item questionnaire with six negative and six positive statement four-point Likert items is one of the most long-standing and extensively used measures of minor psychiatric disorders in community and primary care settings around the world [13]. Despite its widely used, the factor structure of GHQ-12 remains a controversial issue. A simple summed score of GHQ-12 is used initially as a single observed construct of the mental health severity [4, 11]. Table 1 presents the identified GHQ-12's factor structures by the previous studies, along with the study population, applied model, and estimation methods. Some studies suggested unidimensional latent construct with or without adjusting the effects of negative/positive phrased items [14–18]. Some other studies have shown evidence of the scale containing two, three, or four latent factors as multidimensional mental health constructs [19–26]. However, some studies assessing factor structure of GHQ-12 using Exploratory and Confirmatory Factor Analysis (EFA, CFA) and Structural Equation Model (SEM) have ignored Likert-type response data in their parameter estimation methods (assuming the GHQ-12s items as continuous variables follow a normal distribution) [15, 18–20, 23, 27, 28]. A study aimed to compare several coding methods such as original (i.e., 1-2-3-4 scheme for four-response categories), bi-modal (i.e., 0-0-1-1 scheme), and chronic (mixed binary scheme for positively (0-0-1-1) and negatively (0-1-1-1) phrased questions) argued in favor of using original Likert format of GHQs items based on theoretical and empirical reasons [29]. Besides, according to a statistical point of view, a comparative study of multiple parameter estimation approaches concluded that the best one was the ordinal format of items for GHQ-12 factor structure [30]. A recent review article aimed to improve the assessment of health surveys demonstrated useful characteristics of a valuable and flexible model named Multidimensional Graded Response (MGR) model compared to EFA and CFA [31]. The MGR model, as a particular case of Multidimensional Item Response Theory (MIRT), can be used to define multi-component constructs as dimensions of mental health with considering the nature of ordinal responses of GHQ-12s items [31, 32].

**Table 1:** The suggested GHQ-12's factor structures by the previous studies

Studies	Study population	Age range	Applied model	Estimation methods	The best identified model
Gnambs [14]	A representative sample from the population of the United Kingdom or one of its countries	13–100 years	SEM	ML, WLS	Unidimensional factor with adjusting wording effects
Hankins [15]	General population of the UK	Not reported	SEM	ML	Unidimensional factor with adjusting wording effects
Motamed [16]	Adult population of Northern Iran	> = 18 years	CFA	Not reported	Unidimensional factor with correlated errors on negative items
Rodrigo [17]	A representative sample of all employees living in Catalonia (Spain)	17–82 years	CFA	WLS	Unidimensional factor with adjusting wording effects
Smith [18]	A representative sample of the ageing population of the UK	> 50 years	CFA	ML	Unidimensional factor with adjusting negative wording effect
del Pilar Sánchez-López [19]	General Spanish population	25–65 years	EFA	ML	Three-factor structure, including successful coping, self-esteem, and stress
El-Metwally [20]	Al Kharj adult population of Saudi Arabia	> = 18 years	EFA	ML	Three-factor structure, including social dysfunction, anxiety, and loss of confidence
Griffith [21]	A representative UK sample	Mean = 45.8, SD = 18.0	Exploratory SEM	Bayesian estimation and ML	Four-factor structure, including lowered self-worth, social dysfunction, stress, and emotional coping
Mäkikangas [22]	Finnish working-age subjects	25–59 years	CFA	WLS	Three-factor structure, including anxiety/depression, social dysfunction, and loss of confidence
Montazeri [23]	A sample of young Iranian adolescents	18–25 years	EFA	ML	Two-factor structure, including psychological distress and social dysfunction
Najarkolaei [24]	Students of University of Tehran, Iran	18–39 years	EFA and CFA	ML and WLS	Two-factor structure, including social dysfunction and psychological distress
Namjoo [25]	Iranian elder people	> = 60 years	CFA	WLS	Two-factor structure, including social dysfunction and psychological distress
Salama-Younes [26]	Elderly French people	58–72 years	CFA	Not reported	Three-factor structure, including anxiety and depression, social dysfunction and loss of confidence
Gao [27]	A consecutive sample of outpatients with anxiety disorders and/or depressive disorders in Singapore	Not reported	CFA	ML	Three-factor structure, including anxiety and depression, social dysfunction and loss of confidence
Liang [28]	Young Chinese civil servants	20–45 years	EFA and CFA	ML	All one-, two-, and three-dimensional models were well fitted
Smith [49]	Cancer patients with heterogeneous diagnoses in the UK	Late middle age with mean = 57.42	IRT, CFA	ML	Unidimensional factor with correlated error terms
Abubakar [50]	A population of Kenyan adults and adolescents	12–60 years	CFA	Not reported	Three-factor structure, including anxiety, social dysfunction and loss of confidence

*SEM* structural equation model, *ML* maximum likelihood, *WLS* weighted least square, *CFA* confirmatory factor analysis, *EFA* exploratory factor analysis, *IRT* item response theory

The aims of the present study were: (1) to evaluate the latent structure of GHQ-12 within an MGR model framework in a large population-based study; (2) to examine how different aspects of QoL are associated with latent dimensions of mental health in the general population. Moreover, these associations were adjusted for a wide range of potential confounding variables such as demographic, lifestyle-related variables, and BMI [3, 6, 7, 33–35].

## Materials and methods

### Study design and participants

Isfahan Cohort Study (ICS) was a longitudinal population-based cohort study including a representative sample of adults aged 35 years old or over in 2001–2011 [36]. Participants were selected using a multi-stage sampling method. Details about the multistage random sampling procedure (based on urban/rural, sex, and age distribution of the community), along with data collection and study design were published previously [37, 38]. This study included Iranian adults 35 years of age or older, mentally competent and not pregnant. After obtaining informed written consent, different questionnaires, including food frequency questionnaire (FFQ), physical activity, demographic status, smoking and medical history, were completed by participants in a face-to-face interview at baseline. Also, participants underwent clinical examination, electrocardiography and laboratory evaluation. Cardiovascular diseases (CVDs) were the primary outcome events considered in the ICS. The subjects who had a previous history of CVD were excluded at the beginning of the study. Remaining participants were biannually followed-up to detect major CVD. In 2013, ICS was considered a master plan for a multi-generation 10-year cohort study named Isfahan Cohort Study 2 (ICS2) [37]. ICS2 was included a sub-sample of ICS (n = 1487) and a new recruited sample (n = 1355) aged 35 years and over, all living in urban and rural areas of Isfahan and Najafabad, who were initially free of CVDs [37]. The study was approved by the Ethics Committee of Isfahan Cardiovascular Research Center, a World Health Organization collaborating center. All participants provided written informed consent to participate in the study. In the current study, of 1355 newly recruited participants in ICS2 started at 2013, data of 1316 new cases, who had complete information on all variables were included for data analysis.

### Study instruments and data collection

#### Assessment of sociodemographic characteristics

Trained nurses and physicians performed an interview in 2013 to identify the participants’ sociodemographic characteristics, including sex (female/male), age (years), marital status (married/single, divorced, widowed)), place of residence (rural/urban), and educational attainment years (0–5 year/6–12 years/ > 12 years).

### Assessment of lifestyle-related variables

Information on lifestyle behaviors, including dietary behaviors, physical activity, coping strategies and smoking status (ever (current and past) /never) were collected through face-to-face interviews. Moreover, body mass index (BMI) was defined as weight (kg)/ height^2^ (m)). In the current study, we used the International Physical Activity Questionnaire (IPAQ), whose reliability and validity findings were previously published [39, 40]. The total score of an individual's physical activity (expressed as metabolic equivalent task minutes per day (MET-m/d)) consisted of four fields, including homework, leisure time, worksite, and transportation. We categorized total physical activity based on its tertiles in our analysis. To assess adaptive and maladaptive coping strategies validated stress management questionnaire was used [41]. Every one of 30 items is responded to a three-point scale (never, sometimes, and often). For scoring in each individual, the number of items answered “often” is divided by the number of items answered “often” and “sometimes” and is considered the percentage of maladaptive and adaptive skills.

Dietary intake information was evaluated with a validated qualitative, 48-item food frequency questionnaire (FFQ) [42]. Participants answered the frequency of consumption of each item in FFQ in the previous year. Diet quality index (DQI) focused on seven food groups, including fish, soy protein, chicken, or legumes; animal fats, ghee, butter, or hydrogenated oil; fast food; sweets; whole dairy products, meat, or egg; fruit and vegetables; and non-hydrogenated and olive oil. Individual responses of the frequency of consumption in seven food groups were categorized as 2, 1, or 0, in which a higher score reflected a lower nutritional value. The DQI's total score was computed by summing the responses dividing by the number of items. Higher scores of DQI (ranged from 0 to 2) indicate low or unhealthy diet quality. The DQI was previously described in detail [43].

### Assessment of mental health

GHQ-12, as a brief and self-administered questionnaire, was used to evaluate mental health and short-term changes in mental health. Every one of its 12 items is responded to a four-point Likert scale. It consists of six negatives statement items (with response scale including not at all; no more than usual; rather more than usual and much more than usual) and six positive statement items (with a response scale including more than usual; same as usual; less than usual and much less than usual as response scale). Higher values of all items indicate worse mental health. For each item in the GHQ-12, the participants were asked to rate the extent to which they have experienced a symptom during the last week. When the scoring scheme was bimodal (0-0-1-1), the sum score of GHQ-12 was ranged from 0 to 12. Scoring 4 or more is indicated mental health problem. The GHQ-12 was validated in Iran [23].

### Assessment of quality of life

QoL was measured by the WHO Quality of Life—brief version (WHOQOL-BREF), which is a 26-item self-administered questionnaire with a 5-point Likert score format [44]. WHOQOL-BREF highlighted the multidimensional nature of QoL. The first two questions include perceived overall health and perceived overall QoL. The remaining 24 questions assess environmental (8 items), social (3 items), psychological (6 items), and physical (7 items) domains. The first two items on the global rating of QOL and perceived global health are not contained in mentioned four domains, so a sum of 26 items was used to make the overall QoL score. To easily compare the scores between domains, transformed domains and overall scores ranging from 4 to 20 were obtained from row scores (a simple summed score). Domain scores were calculated in a positive direction (higher scores indicate higher QoL). WHOQOL-BREF demonstrated good psychometric values in Iranian populations [45, 46].

### Statistical analysis

Data were analyzed using the R statistical software version 3.6.2 (R Core Team, 2019). P-value < 0.05 was considered statistically significant. Quantitative variables were summarized by the mean ± standard deviation (SD), and qualitative variables were expressed as absolute frequencies (percentages). The independent two-sample t-test was used to compare the mean QoL score and its domains between dichotomous variables (gender, marital status, place of residence, categories of GHQ-12, and smoking status). In addition, we conducted the ANOVA test to determine the differences among polytomous variables, including age groups, education levels, and tertiles of physical activity. When data do not meet the parametric test assumptions, the Mann‐Whitney U and Kruskal–Wallis tests were used, respectively. The univariate associations between domains and overall scores of QoL and quantitative variables were tested by Pearson correlation (or Spearman correlation, if required). In addition, correlation coefficients of latent dimensions and individual GHQ-12’s items were obtained from the Spearman correlation test.

In line with our study first objective, to evaluate the dimensionality or latent structure of GHQ-12, different competing exploratory MIRT models were fitted. MIRTs include a broad family of models, depending on the items’ scale (ordinal, dichotomous, nominal, and so on). In the current analysis, twelve items of GHQ-12 were considered as ordinal variables with four response categories. An MGR model, as one of the MIRT models for ordered categorical items, was used to analyze the data via mirt package (version 1.32.1, April 2020) [32]. Moreover, the Oblimin rotation method was used to achieve more interpretable factors. Identified latent constructs were contained non-zero cross-loading items as an inherent issue in mental measurements [21]. It means that each item of GHQ-12 could be related to more than one latent construct. The extracted latent constructs were labeled and interpreted as mental health dimensions based on the majority of items loaded on, considering a loading >  = 0.20 for retention. Identified latent constructs as continuous variables quantify the extent to which an individual possesses mental health related to dimensions of GHQ-12.

The goodness of the fitted models was assessed by Root Mean Squared Error Approximation (RMSEA) with 90% confidence interval (90% CI), Standardized Root Mean Square Residuals (SRMSR), Comparative Fit Index (CFI), Bayesian Information Criterion (BIC), Akaike Information Criterion (AIC), Tucker-Lewis Index (TLI), corrected AIC (AICc), sample-Size Adjusted BIC (SABIC), and also chi-square (χ2), related degree of freedoms (df), and Log-likelihood. In general, smaller values of AIC, BIC, AICc, SABIC, RMSEA, and SRMSR, as well as bigger values of TLI and CFI, usually show better fit models. The thresholds RSMEA < 0.08, SRMR < 0.08, CFI ≥ 0.95, and TLI ≥ 0.95 were considered model fit satisfactory [47]. In addition to goodness-of-fit indices, it has been suggested that the principle of parsimony (the lower number of parameters) and conceptual grounds to be considered for model selection [48]. The factor scores of the extracted domains of GHQ-12 derived from the best MGR model were computed, as the scores of mental health's dimensions, for using in the regression analysis.

To examine the simultaneous association between all QoL domains and extracted constructs of mental health, simple or multiple multivariate linear regression was performed. In these regression models, the QoL domains were treated as multivariate response variables, and each latent dimension of mental health was considered as the independent variable. Furthermore, simple or multiple univariate linear regression was used to evaluate the relationship between the QoL’s overall score and each dimension of mental health. Crude and adjusted standardized along with unstandardized regression coefficients and 95% CI as the associations’ measures were presented. In adjusted regression models, the confounding effects of demographic variables, including age, sex, educational level, place of residence, and marital status, were considered in the first model. We further adjusted the confounding effects of lifestyle-related variables, including DQI, physical activity, smoking status, and adaptive and maladaptive coping strategies of stress in the second model. Additional adjustments were made for BMI in the final model.

## Results

Table 2 presents descriptive characteristics of study participants.. 49.4% of participants were female. Mean ± SD age of 1316 participants was 56.44 ± 10.78 years (56.18 ± 10.46 in the female and 56.69 ± 11.09 in the male). Of the participants, 31.4%, 47.7%, and 20.9% were 35–49 years, 50–64 years, and ≥ 65 years old. The majority (87.6%) of the participants were married, and about 82% were from urban areas. More details of demographic characteristics and lifestyle variables are shown in Table 2.

**Table 2:** The demographic and life style futures of study participants and their association with domains and overall scores of quality of life

		Quality of life (QoL)				
		Physical health domain	Psychological domain	Social relationships domain	Environment domain	Overall score
Characteristics	Total	15.28 ± 2.92	14.31 ± 2.62	15.02 ± 3.19	14.84 ± 2.78	14.80 ± 2.43
Age categories						
35–49 years	413 (31.4%)	15.80 ± 2.60	14.28 ± 2.44	15.11 ± 3.14	14.87 ± 2.61	14.96 ± 2.22
50–64 years	628 (47.7%)	15.34 ± 2.96	14.36 ± 2.65	15.14 ± 3.17	14.91 ± 2.81	14.87 ± 2.45
≥ 65 years	275 (20.9%)	14.38 ± 3.08	14.22 ± 2.78	14.61 ± 3.3	14.65 ± 2.92	14.43 ± 2.63
P-value^1^	–	< 0.001	0.8	0.03	0.33	0.009
Sex						
Female	650 (49.4%)	14.68 ± 2.94	13.82 ± 2.55	14.72 ± 3.15	14.63 ± 2.77	14.41 ± 2.38
Male	666 (50.6%)	15.86 ± 2.78	14.78 ± 2.59	15.32 ± 3.21	15.04 ± 2.77	15.19 ± 2.41
P-value^2^	–	< 0.001	< 0.001	0.001	0.007	< 0.001
Marital status						
Married	1153 (87.6%)	15.45 ± 2.85	14.45 ± 2.57	15.16 ± 3.13	14.96 ± 2.71	14.95 ± 2.38
Single	163 (12.4%)	14.08 ± 3.14	13.3 ± 2.71	14.06 ± 3.46	13.97 ± 3.04	13.78 ± 2.54
P-value^2^	–	< 0.001	< 0.001	< 0.001	< 0.001	< 0.001
Educational attainment years						
0–5 years	669 (40.4%)	14.49 ± 2.94	13.86 ± 2.69	14.47 ± 3.19	14.25 ± 2.68	14.22 ± 2.42
6–12 years	456 (34.7%)	15.96 ± 2.74	14.64 ± 2.47	15.45 ± 3.14	15.38 ± 2.75	15.29 ± 2.33
> 12 years	191 (14.5%)	16.41 ± 2.49	15.07 ± 2.4	15.93 ± 3	15.65 ± 2.72	15.69 ± 2.14
P-value^1^	–	< 0.001	< 0.001	< 0.001	< 0.001	< 0.001
Place of residence						
Urban	1078 (81.9%)	15.36 ± 2.91	14.35 ± 2.6	15.06 ± 3.2	14.94 ± 2.82	14.86 ± 2.44
Rural	238 (18.1%)	14.93 ± 2.96	14.11 ± 2.69	14.87 ± 3.17	14.41 ± 2.52	14.54 ± 2.37
P-value^2^	–	0.049	0.22	0.4	0.02	0.08
Smoking status						
Ever	182 (14.2%)	15.23 ± 2.92	14.28 ± 2.56	15.02 ± 3.18	14.83 ± 2.78	14.78 ± 2.41
Never	1100 (85.8%)	15.71 ± 2.85	14.55 ± 2.93	15.02 ± 3.36	14.88 ± 2.78	14.99 ± 2.47
P-value^2^	–	0.04	0.051	0.81	0.67	0.18
Physical activity (METs-m/d)						
Tertile 1 (< 480	170.19 ± 178.13	15.27 ± 3.32	14.4 ± 2.85	15.27 ± 3.47	15.23 ± 3.02	14.97 ± 2.68
Tertile 2 (480–1680)	1074.77 ± 354.95	15.36 ± 2.78	14.28 ± 2.55	15.04 ± 3.1	14.83 ± 2.69	14.82 ± 2.39
Tertile 3 (> 1680)	4482.43 ± 4205.32	15.21 ± 2.58	14.22 ± 2.4	14.73 ± 2.95	14.43 ± 2.51	14.61 ± 2.15
P-value^1^	–	0.59	0.21	0.06	< 0.001	0.04
Categories of GHQ-12						
Normal (0–3)	980 (74.5%)	15.77 ± 2.72	14.8 ± 2.44	15.46 ± 3.02	15.19 ± 2.64	15.24 ± 2.26
Mental health problem (4–12)	336 (25.5%)	13.84 ± 3.01	12.88 ± 2.59	13.74 ± 3.34	13.81 ± 2.92	13.53 ± 2.45
P-value^2^	–	< 0.001	< 0.001	< 0.001	< 0.001	< 0.001
Age (years)	56.44 ± 10.78	− 0.17^4^	− 0.01^4^	− 0.06^4^	− 0.04^4^	− 0.08^4^
P-value^3^	–	< 0.001	0.58	0.02	0.19	0.002
Body mass index	27.87 ± 4.53	− 0.09^4^	− 0.06^4^	− 0.08^4^	− 0.05^4^	− 0.07^4^
P-value^3^	–	0.001	0.046	0.006	0.10	0.008
Diet quality index	0.69 ± 0.26	− 0.08^4^	− 0.13^4^	− 0.08^4^	− 0.15^4^	− 0.14^4^
P-value^3^	–	0.004	< 0.001	0.006	< 0.001	< 0.001
Adaptive coping strategy	42.54 ± 31.74	0.08^4^	0.11^4^	0.11^4^	0.05^4^	0.11^4^
P-value^3^	–	0.003	< 0.001	< 0.001	0.07	< 0.001
Maladaptive coping strategy	38.65 ± 38.27	0.02^4^	0.02^4^	0.01^4^	− 0.02^4^	0.01^4^0.01^4^
P-value^3^	–	0.48	0.56	0.83	0.5	0.73
Sum score of GHQ-12	2.33 ± 3.12	− 0.33^4^	− 0.36^4^	− 0.27^4^	− 0.25^4^–0.25^4^	− 0.36^4^
P-value^3^	–	< 0.001	< 0.001	< 0.001	< 0.001	< 0.001

*METs-m/d* metabolic equivalent task minutes per day, *GHQ* General Health Questionnaire; Quantitative variables were expressed as Mean ± SD; Qualitative variables were summarized as number (percentage); Superscripts 1, 2, and 3 indicate: p-values were obtained from ANOVA (or Kruskal Wallis test, if required), independent two-sample t-test (or Mann–Whitney test, if required), and Pearson Correlation (or Spearman Correlation, if required), respectively. Superscript 4 reflects the correlation coefficient between each variable with overall and domains scores of QoL

### The demographic and lifestyle futures of study participants and their association with domains and overall scores of quality of life

The domains and overall scores of QoL at different levels of the demographic and life style features of study participants were shown in Table 2. The mean scores of the QoL domains (ranged 4–20) were; physical (15.28 ± 2.92), psychological (14.31 ± 2.62), environmental (14.84 ± 2.78), and social (15.02 ± 3.19). The mean QoL overall score (ranged 4–20) was 14.80 ± 2.43. 25.5% of the current sample were reflected a high level of mental health problem based on scoring four or more of the GHQ sum score. The domains and overall mean scores of QoL were significantly lower in women, singles, people with lower levels of education, and higher levels of distress.

According to the correlational results represented in Table 2, we found out that a higher QoL overall score was associated with younger age, lower BMI, less mental health problem score, healthier diet quality, and a more adaptive coping strategy. More details of significant differences across demographic and lifestyle variables are shown in Table 2.

### Extraction of latent dimensions of mental health measured by GHQ-12

Bar charts of the response distributions for positively and negatively phrased items of GHQ-12 were provided in Additional File 1: Fig. S1. Responses were given on a different four-point Likert scale for the negative and positive statement items. As shown in Additional File 1: Fig. S1, category 4 for every GHQ-12 item was reported by a minority of people, while the highest frequencies were obtained for category 2 (except for item 12). Graphs show the differential response patterning of positively and negatively worded items in the GHQ-12.

Table 3 presents the factor loadings of the fitted competing exploratory models for extracting the dimensions of mental health based on GHQ-12. In the present study, five different MGR models were specified and labeled based on the majority of items loaded on each factor and existing names from the literature. The competing models were as follows: (1) One-Dimensional model with 12 items loaded on one factor as mental health; (2) Bifactor model with one general factor as mental health and two additional uncorrelated factors (two specific factors) associated with negatively and positively phrased items for adjusting wording effects. In the Bifactor model, each item was influenced by both the general and specific factors. Specific factors cover the remaining common variance left after accounting for the general factor. It means that specific factors showed some features of negatively and positively phrased items that were not captured by the general factor. According to our model results, the general factor explained 39% of the common variance. However, 24% and 9% of the remaining shared variance were explained by positive and negative statement items, respectively. (3) Two-Dimensional model in which twelve items were loaded on two constructs. In the exploratory model, six negative statement items were loaded on one factor, and six positive statement items were loaded on another construct; (4) Three-Dimensional model with considering three latent constructs of twelve items; and finally, Four-Dimensional model in which the six negatively phrased items constructed the first two factors (F1 and F2), whereas the other two factors (F3 and F4) are made up of the six positive statement items. In the model, items 10 (across F1 and F2), 2 and 3 (across F3 and F4) were cross-loading items. The last three mentioned models were correlated trait MGR models in which extracted factors were correlated together.

**Table 3:** Rotated factor loadings of exploratory Multidimensional Graded Response (MGR) models for constructing mental health dimensions measured by Genera Health Questionnaire-12 (GHQ-12)

Items		One-dimensional model	Bifactor model			Two-dimensional model		Three-dimensional model			Four-dimensional model			
			General factor	Specific factor 1	Specific factor 2	F1	F2	F1	F2	F3	F1	F2	F3	F4
GHQ1	Been able to concentrate on whatever you are doing	0.48	0.32	0.50	0.00	− 0.13	− 0.53	0.22	− 0.61	0.30	0.08	0.19	− 0.02	0.65
GHQ2	Felt that you are playing a useful part in things	0.59	0.40	0.70	0.00	0.02	− 0.82	− 0.02	− 0.82	− 0.03	− 0.17	− 0.12	0.23	0.59
GHQ3	Enjoyed normal day-to-day activities	0.64	0.45	0.69	0.00	− 0.06	− 0.80	− 0.02	− 0.81	0.04	− 0.12	0.00	0.40	0.41
GHQ4	Been able to face up to your problems	0.60	0.39	0.77	0.00	0.06	− 0.89	− 0.02	− 0.88	− 0.07	0.03	− 0.02	0.89	0.00
GHQ5	Felt capable of making decisions about things	0.66	0.44	0.77	0.00	0.02	− 0.90	− 0.07	− 0.88	− 0.07	− 0.03	− 0.02	0.89	− 0.01
GHQ6	Felt reasonably happy, all things considered	0.65	0.46	0.71	0.00	− 0.07	− 0.81	− 0.02	− 0.82	0.05	0.03	0.11	0.80	0.04
GHQ7	Lost much sleep over worry	0.63	0.53	0.00	0.48	− 0.74	0.09	− 0.03	0.02	0.71	− 0.02	0.70	− 0.02	0.02
GHQ8	Been feeling unhappy and depressed	0.83	0.70	0.00	0.65	− 0.95	0.08	0.00	− 0.01	0.96	0.02	0.95	− 0.01	0.05
GHQ9	Felt constantly under strain	0.85	0.72	0.00	0.59	− 0.96	0.08	− 0.11	0.00	0.85	− 0.07	0.87	0.06	− 0.03
GHQ10	Felt you could not overcome your difficulties	0.89	0.86	0.00	0.28	− 0.85	− 0.10	− 0.54	− 0.04	0.41	− 0.50	0.44	0.06	0.00
GHQ11	Been losing confidence in yourself	0.88	0.93	0.00	0.04	− 0.76	− 0.19	− 0.90	− 0.01	0.04	− 0.87	0.08	0.01	− 0.01
GHQ12	Thinking of yourself as a worthless person	0.80	0.90	0.00	− 0.10	− 0.66	− 0.20	− 0.85	− 0.07	− 0.001	− 0.92	− 0.05	− 0.01	0.03

The positively phrased items were GHQ1 to GHQ6 (with a response scale including more than usual; same as usual; less than usual and much less than usual)

The negatively phrased items were GHQ7 to GHQ12 (with a response scale including not at all; no more than usual; rather more than usual and much more than usual). See Table 4 in the manuscript for information on goodness of fit indices for all five competing models

The results of the goodness of fit indices for the five models compared are presented in Table 4. The One-Dimensional model had a worst fit than other competing models. However, wording effects adjustment in the One-Dimensional model (as the Bifactor model) considerably improved the goodness of fit indices. The best the goodness of fit indices belonged to Three-Dimensional model with SRMR = 0.033, CFI = 0.996, TLI = 0.979 and RMSEA = 0.051 (95% CI 0.036, 0.068), and Four-Dimensional model with SRMR = 0.028, CFI = 0.994, TLI = 0.984, and RMSEA = 0.051 (95% CI 0.042, 0.061) in our data. According to fit indices, these two best models were not greatly distinguishable. Therefore, the Three-Dimensional model was considered as the best fitted model in our data due to more parsimonious specifications and more interpretable factors.

**Table 4:** Model fit indices for all five competing exploratory Multidimensional Graded Response (MGR) models

MGR Model	${\chi }^{2}$	df	RMSEA (90% CI)	SRMSR	TLI	CFI	Log-likelihood	AIC	AICc	BIC	SABIC
1	193.40	30	0.064 (0.056, 0.073)	0.157	0.967	0.977	− 13,033.42	26,162.85	26,166.56	26,411.6	26,259.13
2	100.46	18	0.059 (0.048, 0.071)	0.036	0.972	0.988	− 11,829.06	23,778.11	23,783.95	24,089.05	23,898.46
3	66.87	19	0.044 (0.033, 0.055)	0.046	0.985	0.993	− 12,006.31	24,130.63	24,136.27	24,436.39	24,248.97
4	40.27	9	0.051 (0.036, 0.068)	0.033	0.979	0.996	− 11,886.86	23,911.72	23,919.47	24,269.3	24,050.12
5	107.13	24	0.051 (0.042, 0.061)	0.028	0.984	0.994	− 11,826	23,807.99	23,817.95	24,212.21	23,964.44

*df* degree of freedom, *RMSEA* root mean squared error approximation, *CI* confidence interval, *SRMSR* Standardized Root Mean Square Residuals, *TLI* Tucker–Lewis Index, *CFI* Comparative Fit Index, *AIC* Akaike Information Criterion, *BIC* Bayesian Information Criterion, *AICc* corrected AIC, *SABIC* sample-Size Adjusted BIC; Model 1: one-dimensional model; Model 2: Bifactor model; Model3: two-dimensional model; Model 4: three-dimensional model; Model 5: four-dimensional model

**Fig. 1:**
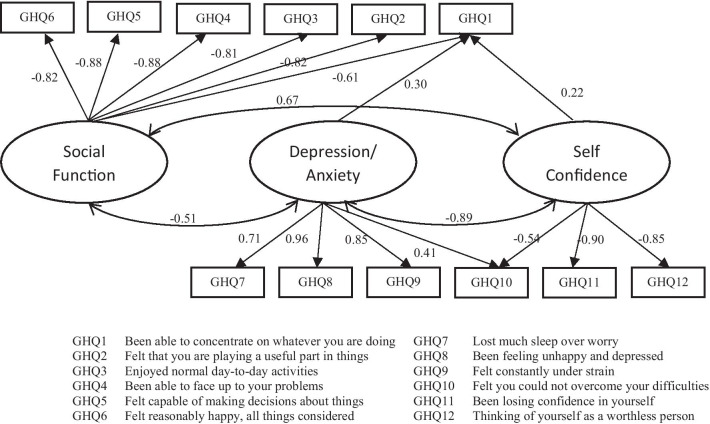
Graphical presentation of three-dimensional graded response model for 12-item General Health Questionnaire in the present study

In Table 3, the first latent construct (F1), namely Self Confidence (SC) of the Three-Dimensional model, as the best MGR model, was characterized by items 1, 10, 11, and 12 from GHQ-12. The second latent construct, labeled as Social Function (SF), contained items 1 to 6 (positively worded items). The third one, named Anxiety/Depression (A/D), was considerably loaded with items 1, 7, 8, 9, and 10. As shown in Table 3, items 1 and 10 were cross-loading items and loaded on multiple underlying constructs. Item 1 was loaded on all constructs, while item 10 was loaded on the dimensions of SC and A/D. Moreover, the correlation between the dimensions of SC and SF was 0.67, between the dimensions of SC and A/D was − 0.89, and also between the dimensions of SF and A/D was − 0.51. Furthermore, Cronbach’s alpha coefficients were obtained between 0.76 and 0.85 for three latent constructs of the Three-Dimensional model. Figure 1 showed graphical presentation of the Three-Dimensional model, as the best MGR model, for GHQ-12 in the current study.

Mean and 95% CI of latent constructs of mental health based on Three-Dimensional model were presented across their constituent items using Error-Bar charts in Additional File 1: Fig. S2. Furthermore, Additional File 1: Fig. S2 graphically depicts the mean and 95% CI of three latent dimensions across sum score of GHQ (ranged 0 to 12). Lower values of all twelve items and the sum score of GHQ reflect better mental health and vice versa. Higher values of the SC and SF dimensions indicate the lower intensity of mental health, while higher scores of the A/D reflect more elevated mental health. As shown in Additional File 1: Fig. S2, the intensity of mental health dimensions was different across various levels of GHQ sum scores, in particular with scoring four or more as high distress level.

Furthermore, the correlation coefficients between GHQ-12’s latent dimensions and domains of QOL were presented in Additional File 1: Table S1. Significant negative correlations between all domains of QOL and the A/D dimension of mental health revealed an acceptable divergent Validity. In addition, SC and SF dimensions of mental health had moderate positive correlations with all domains of the QOL, ranging from 0.24 to 0.39 (p < 0.001), indicating satisfactory convergent validity based on association analysis with QOL.

Moreover, we evaluated the discriminant validity of newly extracted domains of mental health internally (Additional File 1: Table S1). Findings of item-scale correlations (with overlap correction) showed a higher correlation between each item with its related factor. Moreover, a lower correlation was found between each item with other factors.

### The relationship between latent dimensions of mental health and quality of life domains

Table 5 shows the results of crude and adjusted regression models of the relationships between domains of QoL with dimensions of mental health, and the sum score of GHQ. In the crude model, we reached significant positive associations of SC and SF dimensions of mental health with all aspects of QoL. Furthermore, higher scores of the A/D dimension (worse psychological health) were associated with lower scores of all aspects and the overall QoL. The mentioned associations of mental health dimensions in later models were slightly changed by further adjustment for other confounders, but these associations remained enormously significant.

**Table 5:** Crude and adjusted regression coefficients of the association of new-extracted mental health dimensions as predictors and QoL domains as dependent variables

Mental health dimensions	Model	Quality of life (QoL)									
		Physical health domain		Psychological domain		Social relationships domain		Environment domain		Overall	
		Unstandardized coefficients (95% CI)	Standardized coefficients	Unstandardized coefficients (95% CI)	Standardized coefficients	Unstandardized coefficients (95% CI)	Standardized coefficients	Unstandardized coefficients (95% CI)	Standardized coefficients	Unstandardized coefficients (95% CI)	Standardized coefficients
Self confidence dimension	1	1.18 (1.07, 1.30)	0.48	1.18 (1.08, 1.28)	0.54	1.16 (1.03, 1.29)	0.43	1.02 (0.90, 1.13)	0.44	1.12 (1.03, 1.21)	0.55
	2	1.07 (0.96, 1.19)	0.44	1.12 (1.01, 1.22)	0.51	1.11 (0.97, 1.25)	0.41	0.97 (0.85, 1.08)	0.42	1.05 (0.96, 1.15)	0.52
	3	1.05 (0.93, 1.17)	0.43	1.06 (0.95, 1.17)	0.49	1.03 (0.89, 1.17)	0.39	0.91 (0.79, 1.03)	0.39	0.99 (0.89, 1.09)	0.49
	4	1.05 (0.92, 1.17)	0.43	1.04 (0.93, 1.15)	0.48	1.02 (0.87, 1.16)	0.38	0.89 (0.77, 1.01)	0.39	0.98 (0.88, 1.08)	0.49
Social function dimension	1	0.86 (0.74, 0.98)	0.37	0.93 (0.83, 1.03)	0.45	0.88 (0.75, 1.01)	0.35	0.71 (0.59, 0.83)	0.32	0.83 (0.74, 0.93)	0.43
	2	0.74 (0.62, 0.85)	0.32	0.86 (0.76, 0.96)	0.41	0.81 (0.68, 0.94)	0.32	0.64 (0.52, 0.75)	0.29	0.75 (0.66, 0.85)	0.39
	3	0.73 (0.61, 0.84)	0.31	0.82 (0.71, 0.92)	0.39	0.76 (0.62, 0.89)	0.30	0.62 (0.50, 0.73)	0.28	0.71 (0.61, 0.81)	0.37
	4	0.69 (0.58, 0.82)	0.30	0.79 (0.68, 0.89)	0.38	0.73 (0.59, 0.86)	0.29	0.58 (0.46, 0.70)	0.27	0.68 (0.58, 0.78)	0.36
Anxiety-depression dimension	1	− 1.03 (− 1.15, − 0.92)	− 0.43	− 1.01 (− 1.11, − 0.91)	− 0.47	− 0.93 (− 1.07, − 0.80)	− 0.36	− 0.79 (− 0.91, − 0.68)	− 0.35	− 0.93 (01.03, − 0.84)	− 0.47
	2	− 0.95 (− 1.06, − 0.83)	− 0.39	− 0.94 (− 1.05, − 0.83)	− 0.44	− 0.88 (− 1.02, − 0.74)	− 0.34	− 0.75 (− 0.86, 0.63)	− 0.33	− 0.87 (− 0.96, − 0.77)	− 0.41
	3	− 0.91 (− 1.03, − 0.79)	− 0.38	− 0.89 (− 0.99, − 0.78)	− 0.41	− 0.82 (− 0.96, − 0.68)	− 0.31	− 0.69 (− 0.81, − 0.56	− 0.30	− 0.81 (− 0.91, − 0.71)	− 0.41
	4	− 0.91 (− 1.03, − 0.78)	− 0.38	− 0.86 (− 0.97, − 0.75)	− 0.40	− 0.80 (− 0.95, − 0.66)	− 0.31	− 0.67 (− 0.79, − 0.54)	− 0.29	− 0.79 (− 0.89, − 0.69)	− 0.4
Sum score of GHQ-12	1	− 0.34 (− 0.38, − 0.29)	− 0.36	− 0.33 (− 0.37, − 0.29)	− 0.39	− 0.31 (− 0.37, − 0.26)	− 0.31	− 0.26 (− 0.31, − 0.21)	− 0.29	− 0.31 (− 0.34, − 0.27)	− 0.39
	2	− 0.29 (− 0.34, − 0.24)	− 0.31	− 0.29 (− 0.34, − 0.25)	− 0.35	− 0.28 (− 0.34, − 0.23)	− 0.28	− 0.23 (− 0.28, − 0.18)	− 0.26	− 0.27 (− 0.31, − 0.23)	− 0.35
	3	− 0.29 (− 0.34, − 0.25)	− 0.32	− 0.30 (− 0.34, − 0.26)	− 0.36	− 0.29 (− 0.34, − 0.23)	− 0.28	− 0.23 (− 0.28, − 0.18)	− 0.26	− 0.27 (− 0.31. − 0.23)	− 0.35
	4	− 0.29 (− 0.33, − 0.24)	− 0.31	− 0.29 (− 0.34, − 0.25)	− 0.35	− 0.27 (− 0.33, − 0.22)	− 0.27	− 0.22 (− 0.26, − 0.17)	− 0.24	− 0.26 (− 0.30, − 0.22)	− 0.34

Multivariate (simple or multiple) linear regression was used for evaluation the association of all domains of QoL with GHQ-12 dimensions and univariate (simple or multiple) linear regression was used for the association of sum score of GHQ-12 with overall QOL score

All P-value < 0.001

Model 1: unadjusted model

Model 2: adjusted for demographic variables (age, sex, education, marital status, area of residence)

Model 3: adjusted for demographic variables and lifestyle-related variables (smoking status, physical activity, diet quality index and coping strategies)

Model 4: adjusted for demographic variables, lifestyle-related variables, and BMI

In the full adjusted model, a one-unit increase in SC dimension was associated with 1.05-, 1.04-, 1.02-, 0.89-, and 0.98-unit increase in the mean scores of physical health, psychological, social relationships, environment domains, and overall score of QoL, respectively. Furthermore, standardized coefficients suggested that a one-SD increase in SC dimension was associated with 43%, 48%, 38%, 39%, and 49% -SD increase in physical health, psychological, social relationships, environment domains and the overall scores of QoL, respectively.

Likewise, after adjusting for confounding variables, a one-SD increase in SF dimension increased the scores of physical health, psychological, social relationships, environment domains, and the overall QoL by more than one-quarter of an SD. Similar to the SC dimension of mental health, the highest to the lowest standardized coefficients of the SF dimension were respectively related to the psychological, physical health, social relationships, and environmental condition domains.

**Fig. 2:**
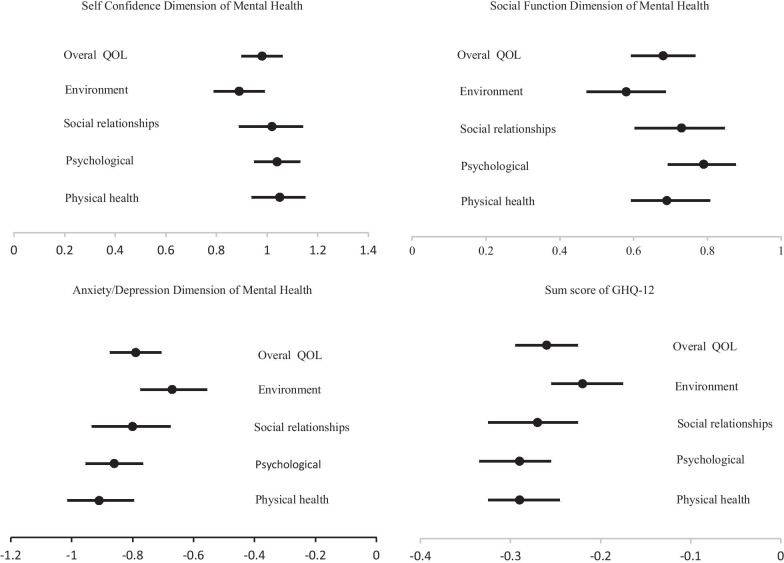
The points in the lines and upper and lower limits depict the adjusted regression coefficients and 95% confidence interval of relationships between dimensions of mental health and quality of life domains

On the contrary, in the full adjusted model, one-SD increase in score of A/D dimension can lead to SD decline of 38%, 40%, 31%, 29%, and 40% in scores of physical health domain, psychological domain, social relationships domain, environment domain and the overall QoL respectively. Likewise, SD decrease of sum score of GHQ was 31%, 35%, 27%, 24%, and 34%, respectively. For the A/D dimension, the psychological domain of QoL had the highest standardized coefficient of association, while the lowest standardized coefficient of the association was found for the environmental condition domain. More details on the relationship between QoL domains with mental health dimensions in other adjusted models can be found in Table 5. Figure 2 graphically depicts the full adjusted unstandardized coefficients and 95% CI of the relationships between dimensions of mental health and QOL domains.

## Discussion

In this study, we explored the latent structures of GHQ-12 as dimensions of mental health within the MGR model framework as a powerful modeling approach evaluating questionnaires with ordered Likert format responses [31] in a large population-based study in Isfahan, Iran. In this regard, we compared five competing exploratory models, including (1) One-Dimensional model with all GHQ-12 items loaded on one latent construct as mental health, (2) Bifactor model with one general factor as mental health, and two specific factors for considering the role of wording effects associated with negatively and positively phrased items; (3) Two-Dimensional model in which six negative statement items were loaded on one factor and six positive statement items were loaded on another construct, (4) Three-Dimensional model identifying three latent constructs of SC (items 1, 10–12), SF (items 1–6), and A/D (items 1, 7–10); and finally, Four-Dimensional model in which the six negatively phrased items constructed the first two factors, whereas the other two factors are made up of the six positive statement items. In the models under consideration, cross-loading items in each identified construct and the paired correlation between latent constructs in each model were considered. Furthermore, after selecting the best-fitted model, we examined how different aspects of QoL, including physical health, psychological, social relationships, environment domains, are associated with latent dimensions of mental health in the general population.

Our findings of the current study showed One-Dimensional model had a worst fit than the alternative models. In agreement with our results, many studies are available in favor of the multidimensional compared to the unidimensional structure of GHQ-12 around the world among various populations such as the employee population [17], diseases-specific populations [28, 49], and adolescents and general populations [16, 22, 50].

Moreover, in disagreement with some studies [14, 16–18], findings of the present study showed although the unidimensional model with adjusting wording effects associated with negatively and positively phrased items considerably improved fit indices compared to the One-Dimensional model, it did not give a suitable fit compared to Three and Four-Dimension models in our population. Following these findings in our study, similar results were observed in some studies [50]. In the current study, although the Four-Dimensional model gave suitable fit indices, the best model for GHQ-12 latent structures was considered the Three-Dimensional model based on the balance of better fit indices, more parsimonious estimation, and more interpretable latent dimensions.

Regardless of the consideration of the ordinal nature of response categories in the parameter estimation method and non-zero cross-loading items of GHQ-12, some previous studies are in favor of the Three-Dimensional model. In this regard, a recent cross-sectional study from the Al Kharj population in Saudi Arabia identified three factors of GHQ-12, including social dysfunction (items 1–6), anxiety (items 7–10), and loss of confidence (items 11–12) using CFA among people aged 18 years and older [20]. In addition, in another study among the general Spanish population aged between 25 and 65 years, a three-factor structure of GHQ-12 was proposed using EFA with cross-loading items but ignoring the ordinal nature of items. They used different names from those used by the current study. Identified three factors, namely Successful Coping (items 1–6), Self-esteem (items 8, 10–12), and Stress (items 7–9), were similar to ours with small differences in the cross-loading items [19]. Furthermore, the Three-Dimensional model also provided a superior fitted level in other previous studies, including Finnish working-age subjects between 25 and 59 years old [22], 58–72 years French adults [26], and clinical cases in Singapore [28].

In contrast with our findings, some studies among the Iranian population in other areas of the country have achieved different latent structures of GHQ-12 among young people aged 18 to 25 years old [23], university students [24], and the elderly population [25]. Findings of the recent study of Northern Iran on participants aged ten years and older revealed the model with the best fit was one-dimensional model adjusting negative statement items effect, followed by the three-dimensional model (social dysfunction (items 1–6), A/D (items 7–10), and loss of confidence (items 11–12)) [16]. In this regard, it was suggested that the underlying latent structure might depend on the population under study [18].

Taken together, some of the inconsistencies of GHQ-12’s factor structure in related studies might be because of the participant’s levels of distress [18]; response bias to positive and, or negative statement items [14]; different parameter estimation approaches used [30]; multiple schemes for scoring the GHQ-12s items [29]; various latent variable modeling for assessing latent constructs such as CFA, EFA, IRT, and SEM; differential item functioning due to individual differences, in particular gender- and age-related differential item functioning [17, 49]; misinterpretation of items in the specific negative statement due to the different level of participants education; with/without considering non-zero cross-loadings; and diverse populations under consideration.

Furthermore, in the second objective of the current study, we examined the associations of identified latent mental health dimensions with all QoL aspects. After adjusting for age, sex, education, marital status, area of residence, smoking status, physical activity, diet quality index, coping strategies, and BMI, we reached significant positive associations of SC and SF dimensions of mental health with all QoL aspects. In other word, a one-SD increase in dimensions of SC and SF was associated with a 38- to 48%-SD and 27- to 38%-SD increase in the domains scores of QoL, respectively. Furthermore, the present study showed higher scores of A/D dimension (worse psychological health) were associated with lower scores of all aspects of QoL. In other word, for each one‐SD increase in score of A/D dimension, the domains scores of QoL decreased by 29- to 40%-SD. The highest to the lowest standardized coefficients for all latent mental health dimensions were respectively related to the psychological, physical health, social relationships, and environmental condition domains of QoL. It means that the psychological aspect of QoL is the most affected by all latent mental health dimensions, followed by physical health, social relationships, and environmental condition domains.

Limited studies have focused on the association of latent structures of mental health and QoL domains. Regardless of latent or obvious structures of assessments, simple or advanced statistical analysis, evaluating separate or simultaneous relationships, and underlying instruments under consideration, our findings conform with previous research supporting that QoL domains are highly associated with the mental health levels among general and specific populations [3–11].

In line with our findings, a cross-sectional study with a small sample size of outpatients with depression and, or anxiety disorders in Singapore showed that the three latent factors of GHQ-12, named social dysfunction (items 1–6), anxiety and depression (items 7–10), and loss of confidence (items 11–12) gave the best fit levels using CFA. They found that diminished all health-related QoL domains (measured by Short Form 36 Health Survey (SF-36)) were considerably associated with three latent factors of mental health. This study, using simple statistical analysis, showed that social dysfunction, anxiety and depression, and loss of confidence were negatively correlated with physical functioning, general health, bodily pain, mental health, social functioning, role limitations due to physical problems, vitality, and role limitations due to emotional problems. In disagreement with our findings, they found that correlation coefficients of three latent factors of mental health were in the neighborhood of 0.83 to 0.90, which it may be difficult to distinguish them [28].

In previous studies, the close link between mental disorders, including depression and anxiety, and almost all QoL domains, as measured by WHOQOL-BREF, are well established [3, 6, 10, 33]. It was shown that reduced QOL was considerably associated with even low levels of depression and anxiety symptoms [3]. A cross-sectional study on primary care patients with common mental disorders (such as depression and anxiety) found out that symptom intensity and comorbidity were significantly associated with a decrease in various aspects of QoL, especially the psychological and physical domains [33]. In line with our results, a cross-national study in six European countries showed that mental disorders have the most impact on the psychological than the physical domain of QoL [34]. Moreover, given the marked significant and independent relationships between latent dimensions of mental health and the social and environmental domains of QoL in the current study, it seems that in an attempt to full recovery as assessed by improved QoL outcomes, treatment of clinical symptoms may not be sufficient, as concluded in previous studies [33]. Several potential mechanisms explain the loss of different aspects of QoL due to dimensions of mental health. Impairments and limitations in cognitive, emotional, and motivational function caused by mental health problems may lead to disability and loss of quality of life [34].

This study is one of the first studies which has simultaneously investigated the association between all domains of QoL and latent dimensions of mental health identified by the MGR model in a representative sample of the general population. Our findings have some limitations and strengths. The current study considered the real nature of items assessing latent structure using a reliable statistical method. We believe that the family of Item Response Theory models can help to improve the quality of developing instruments. However, identifying the latent structure of mental health with considering individual differences is suggested in future studies.

Moreover, future researches may benefit from taking into account extreme response style (systematic tendency to choose extreme or middle options in Likert-type or rating scale items) in latent structures [51]. Because of the cross-sectional nature of the current study, causality and directionality of the effects cannot be determined. However, a large and representative sample in the current study enables us to have adequate power to interpret our results. Also, we used the complete-case method to handle missing data, so more advanced approaches such as multiple imputations are recommended in future studies. Further research would be valuable to consider some likely confounders/mediators such as personality traits, social support, and life stressors which were not in our adjusted models.

## Conclusions

GHQ-12 is a useful screening instrument for mental health status in the community and primary care settings worldwide due to the brevity and easiness of administration and comprehensibility. The current study identified three correlated yet different latent dimensions of GHQ-12, named SF, assessing psychological health; SC, indicating individual’s relationship to himself/herself; and A/D, reflecting psychological disorders using the advanced statistical analysis by focusing on tackling the parameter estimation challenges. Our findings confirm that the GHQ-12 as a multidimensional rather than unitary instrument measures distinct mental health aspects. Furthermore, we appropriately found that all aspects of QoL declined when the intensity of A/D dimensions increased. On the contrary, higher scores of SF and SC dimensions were associated with improved all aspects of QoL. Moreover, the psychological aspect of QoL is the most affected by all latent mental health dimensions, followed by physical health, social relationships, and environmental condition domains. Besides, given the marked significant and independent associations between latent dimensions of mental health and the social and environmental domains of QoL in the current study, it seems that in an attempt to full recovery as assessed by improved QoL outcomes, treatment of clinical symptoms may not be sufficient.

In future studies to establish and distinguish the clinical benefits of mental health dimensions, it is suggested to compare the factor scores in different groups of chronic somatic conditions and mental diseases. Identifying and differentiating the mental health structures in each community and implementing intervention programs focused on specific dimensions may help prevent further deterioration and improve QoL. Other researches are needed to confirm our findings by focusing on overcoming our limitations.

## Supplementary Information

**Additional file 1.** Additional data about items of GHQ-12 and its newly explored domains.

## Data Availability

The data sets used and, or analyzed during the current study are available from the corresponding author on reasonable request.

## Abbreviations

QoL: Quality of life
WHO: World Health Organization
GHQ: General Health Questionnaire
EFA: Exploratory factor analysis
CFA: Confirmatory factor analysis
SEM: Structure equation model
MGR: Multidimensional Graded Response
MIRT: Multidimensional Item Response Theory
ICS: Isfahan Cohort Study
ICS2: Isfahan Cohort Study 2
BMI: Body mass index
MET-m/d: Metabolic equivalent task minutes per day
IPAQ: International Physical Activity Questionnaire
FFQ: Food frequency questionnaire
DQI: Diet quality index
WHOQOL-BREF: WHO Quality of Life-brief version
SD: Standard deviation
RMSEA: Root mean squared error approximation
CI: Confidence interval
SRMSR: Standardized Root Mean Square Residuals
TLI: Tucker–Lewis Index
CFI: Comparative Fit Index
AIC: Akaike Information Criterion
BIC: Bayesian Information Criterion
AICc: Corrected AIC
SABIC: Sample-Size Adjusted BIC
χ^2^: Chi-square
df: Degree of freedoms
SC: Self confidence
SF: Social function
A/D: Anxiety/depression
SF-36: Short Form 36 Health Survey
