# Supplementary material for: How different domains of quality of life are associated with latent dimensions of mental health measured by GHQ-12
Source: Health Qual Life Outcomes. 2021 Nov 14;19:255. doi: 10.1186/s12955-021-01892-9 (PMC8591883; doi:10.1186/s12955-021-01892-9)
Supplement: Supplementary file 1 — Additional file 1. Additional data about items of GHQ-12 and its newly explored domains. [file 12955_2021_1892_MOESM1_ESM.docx]

**Supplementary Figure 1:** Bar charts of the response distributions for positively and negatively phrased items of GHQ-12. Graphs showing the differential response patterning of positive and negative statement items.

**Supplementary Figure 2:** Mean and 95% confidence interval (CI) of latent dimensions of mental health based on Three-Dimensional model across their constituent items and sum score of GHQ. The positively phrased items were GHQ1 to GHQ6 (with response scale including more than usual; same as usual; less than usual and much less than usual as response scale). The negatively phrased items were GHQ7 to GHQ12 (with response scale including not at all; no more than usual; rather more than usual and much more than usual).


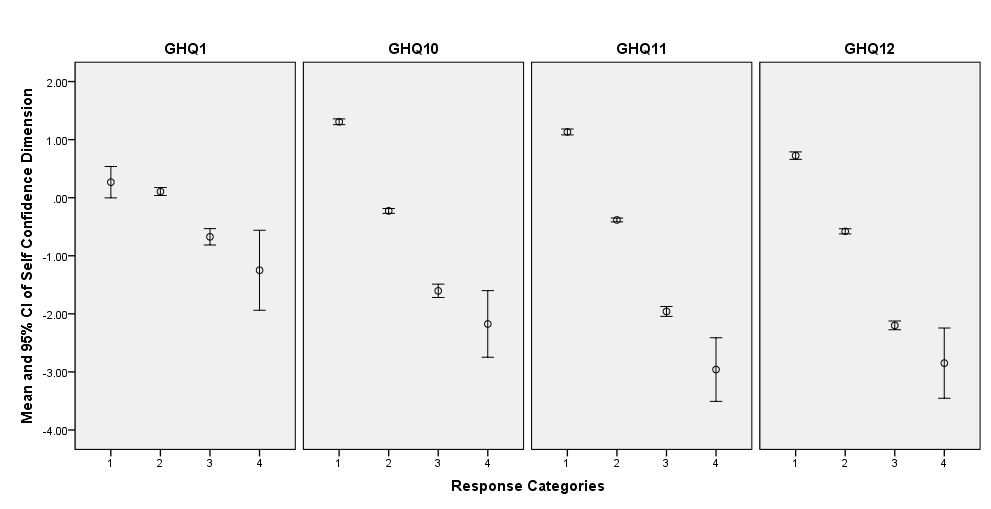


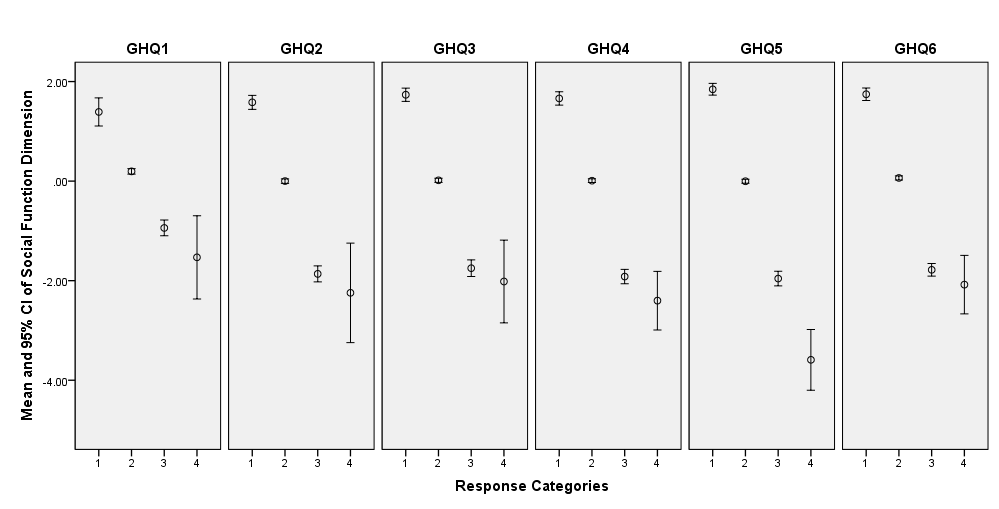


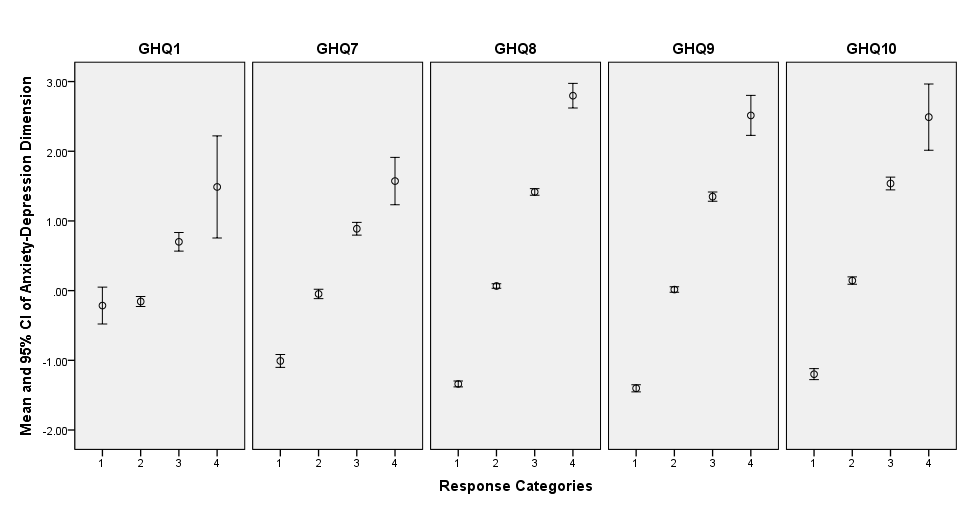


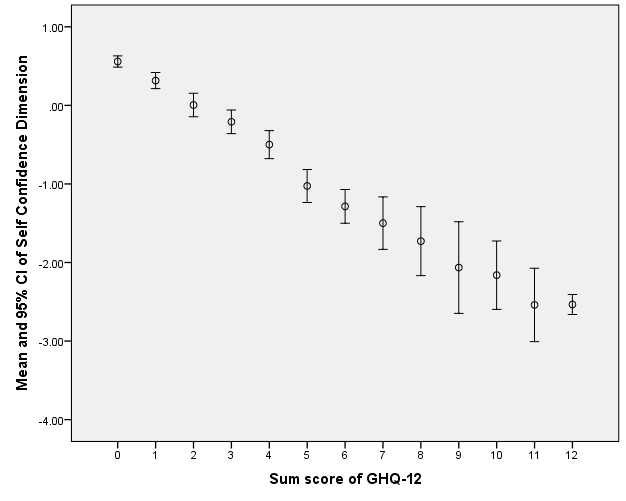

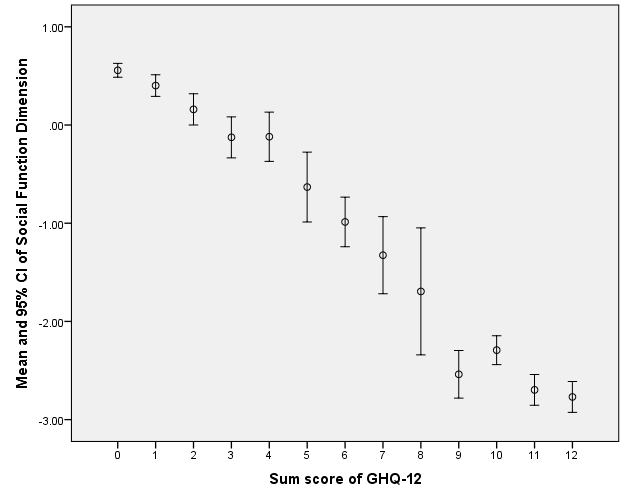

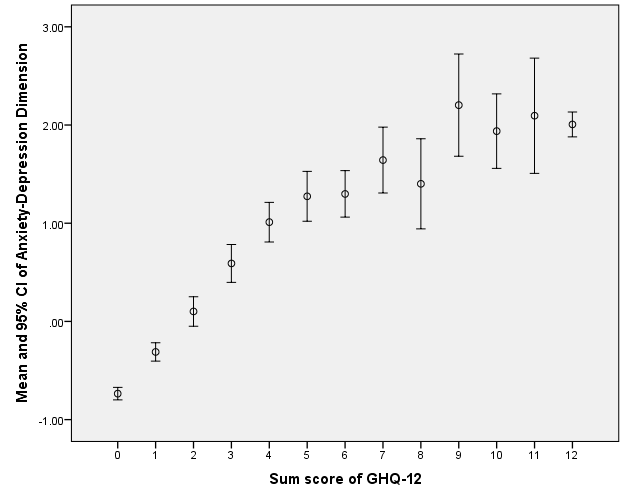


| Items | | Latent dimensions of mental health | | | Item-scale  correlations** |
| --- | --- | --- | --- | --- | --- |
|  |  | Self Confidence | Social Function | Anxiety/Depression |  |
| GHQ1 | Been able to concentrate on whatever you are doing | **-0.28** | **-0.49** | **0.31** | -0.23 ^SC^, -0.45 ^SF^, 0.27 ^A/D^ |
| GHQ2 | Felt that you are playing a useful part in things | -0.39 | **-0.67** | 0.28 | -0.65 ^SF^ |
| GHQ3 | Enjoyed normal day-to-day activities | -0.43 | **-0.67** | 0.33 | -0.67 ^SF^ |
| GHQ4 | Been able to face up to your problems | -0.39 | **-0.71** | 0.27 | -0.68 ^SF^ |
| GHQ5 | Felt capable of making decisions about things | -0.43 | **-0.70** | 0.30 | -0.72 ^SF^ |
| GHQ6 | Felt reasonably happy, all things considered | -0.46 | **-0.73** | 0.37 | -0.67 ^SF^ |
| GHQ7 | Lost much sleep over worry | -0.55 | -0.33 | **0.69** | 0.56 ^A/D^ |
| GHQ8 | Been feeling unhappy and depressed | -0.74 | -0.47 | **0.92** | 0.79 ^A/D^ |
| GHQ9 | Felt constantly under strain | -0.75 | -0.46 | **0.88** | 0.76 ^A/D^ |
| GHQ10 | Felt you could not overcome your difficulties | **-0.81** | -0.56 | **0.77** | -0.67 ^SC^, 0.68 ^A/D^ |
| GHQ11 | Been losing confidence in yourself | **-0.87** | -0.59 | 0.69 | -0.73 ^SC^ |
| GHQ12 | Thinking of yourself as a worthless person | **-0.78** | -0.53 | 0.57 | -0.63 ^SC^ |
| Physical health domain of QOL | | 0.35 | 0.27 | -0.32 | ---- |
| Psychological domain of QOL | | 0.39 | 0.33 | -0.35 | ---- |
| Social relationships domain of QOL | | 0.32 | 0.27 | -0.28 | ---- |
| Environment domain of QOL | | 0.30 | 0.24 | -0.25 | ---- |
| Overall score of QOL | | 0.39 | 0.32 | -0.34 | ---- |

**Supplementary Table 1:** Correlation coefficients between GHQ-12’s latent dimensions and domains of quality of life and GHQ-12’s items

SC: Self Confidence dimension; SF: Social Function dimension; A/D: Anxiety/Depression dimension

All P-values were <0.001.

Correlation coefficients of latent dimensions and individual GHQ-12’s items and quality of life domains were obtained from Spearman's rho test.

** Pearson’s correlation coefficients between each item and its own extracted latent dimension (superscript name) corrected for overlap (with item removed).
